# An ensemble of neural models for nested adverse drug events and medication extraction with subwords

**DOI:** 10.1093/jamia/ocz075

**Published:** 2019-06-14

**Authors:** Meizhi Ju, Nhung T H Nguyen, Makoto Miwa, Sophia Ananiadou

**Affiliations:** 1 National Centre for Text Mining, School of Computer Science, The University of Manchester, Manchester, UK; 2 Toyota Technological Institute, Nagoya, Japan; 3 Artificial Intelligence Research Centre (AIRC), National Institute of Advanced Industrial Science and Technology (AIST), Tokyo, Japan

**Keywords:** adverse drug event, nested named entity recognition, information extraction, natural language processing, electronic health record

## Abstract

**Objective:**

This article describes an ensembling system to automatically extract adverse drug events and drug related entities from clinical narratives, which was developed for the 2018 n2c2 Shared Task Track 2.

**Materials and Methods:**

We designed a neural model to tackle both nested (entities embedded in other entities) and polysemous entities (entities annotated with multiple semantic types) based on MIMIC III discharge summaries. To better represent rare and unknown words in entities, we further tokenized the MIMIC III data set by splitting the words into finer-grained subwords. We finally combined all the models to boost the performance. Additionally, we implemented a featured-based conditional random field model and created an ensemble to combine its predictions with those of the neural model.

**Results:**

Our method achieved 92.78% lenient micro F1-score, with 95.99% lenient precision, and 89.79% lenient recall, respectively. Experimental results showed that combining the predictions of either multiple models, or of a single model with different settings can improve performance.

**Discussion:**

Analysis of the development set showed that our neural models can detect more informative text regions than feature-based conditional random field models. Furthermore, most entity types significantly benefit from subword representation, which also allows us to extract sparse entities, especially nested entities.

**Conclusion:**

The overall results have demonstrated that the ensemble method can accurately recognize entities, including nested and polysemous entities. Additionally, our method can recognize sparse entities by reconsidering the clinical narratives at a finer-grained subword level, rather than at the word level.

## INTRODUCTION

Electronic health records (EHRs)—a digital version of a patient’s information and medical history—are an important source of health data that can impact on a patient’s care. Mining such data would help improve the understanding of treatment and diagnosis of disease.^1^^,^^2^ Among the many known application areas of EHR mining,^2^^,^^3^ adverse drug event detection has been proven to improve and complement drug safety surveillance strategies.

The World Health Organization defines an adverse drug event (ADE) as “an injury resulting from medical intervention related to a drug.”^4^ This work focuses on extracting such ADE mentions and their related medications from EHRs. We base our analysis on data sets provided by the n2c2 Shared Task Track 2, consisting of discharge summaries drawn from the MIMIC-III clinical care database.^5^ This task involves identification of nine entity types: *ADE*, *Dosage*, *Duration*, *Drug*, *Form*, *Frequency*, *Reason*, *Route,* and *Strength*.

Approaches to ADE detection in EHRs are divided into rule-based, machine learning-based and neural network-based categories. Iqbal et al^6^ detected ADEs based on a predefined dictionary and postprocessing rules. Similarly to Iqbal et al,^6^ Yeleswarapu et al^7^ detected drugs and ADEs from multiple data sources using dictionaries compiled from the Medical Subject Headings (MeSH)^8^ and Medical Dictionary for Regulatory Activities (MedDRA),^9^ respectively. Wang et al^10^ proposed a framework to extract vaccine ADEs by combining formal ADE reports from Vaccine Adverse Event Reporting System (VAERS) with ADEs in social media (Twitter) and applying multi-instance learning methods. Nikfarjam et al^11^ also extracted adverse drug reactions from social media by utilizing word embedding cluster features, whereas Korkontzelos et al^12^ used sentiment analysis features.

The Text Analysis Conference (TAC) 2017 Adverse Reaction Extraction from Drug Labels Track^13^ is a similar shared task to the n2c2 Shared Task, but it focuses instead on drug labels. Of the tasks in TAC 2017, one was to recognize six ADE types: adverse reaction, drug class, severity, factor, animal, and negation. The most common approach was the use of bi-directional long short-term memory (BiLSTM) with conditional random fields (CRFs).^14–19^ These systems were implemented with precalculated word embeddings and dynamically learned character embeddings. Several external resources were also used, such as MEDLINE, MedDRA, SIDER, and Unified Medical Language System (UMLS).

The MADE1.0 NLP challenge (http://bio-nlp.org/index.php/announcements/39-nlp-challenges) was another similar shared task, involving detection of mentions of medication names and their attributes (dosage, frequency, route, and duration), as well as mentions of ADEs, indications, and other signs and symptoms in EHRs of cancer patients. Neural network models (eg, long short-term memory (LSTM),^20^ bidirectional long short-term memory (BiLSTM),^21^ and BiLSTM-CRF,^22–24^ were the most popular approaches for ADE detection.

## OBJECTIVE

This study presents an ensemble system to automatically extract ADEs and information about medications from EHRs, based on the 2018 n2c2 Shared Task Track 2. We have focused on subtask 1: the identification of drugs and their attributes, hereafter referred to as entities. Unlike existing models that focus on flat (ie, non-nested) entities,^25–27^ our model can detect both flat and nested entities (entities embedded in other entities) including polysemous entities (ie entities annotated with multiple semantic types), without depending on any external knowledge resources or hand-crafted linguistic features.^28^ To improve the extraction of sparse entities, we further incorporated subwords using byte pair encoding.^29^ To take advantage of feature-based models, we additionally implemented a CRF model with token-based features, dictionary features, and cluster features. We created two types of ensemble using majority voting^30^: (1) intra-ensemble that combines different versions of the same model with different parameter settings, and (2) inter-ensemble, which combines different models or different intra-ensembles. We refer to the models with intra- and inter-ensemble settings as intra-model and inter-model, respectively.

Experimental results showed that, in most cases, the CRF model produced better precision, whereas the neural network (NN) model attained higher recall. The combination of these two models yielded the highest lenient recall on the development set, whereas the best lenient F-score was produced by an inter-ensemble of the NN models. We show that our ensemble neural models using subwords are effective not only in extracting nested entities but also in improving the recognition of sparse entities, achieving 92.78% micro F-score with 95.99% precision and 89.78% recall on the test set in terms of lenient matching.

## MATERIALS AND METHODS

**Figure 1.**
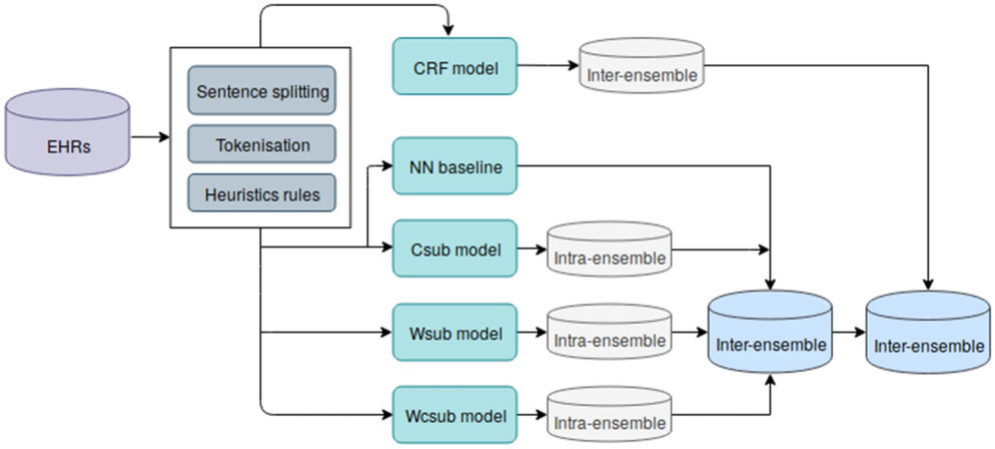
An overview of our work. Intra-ensemble refers to the combination of different versions of the same model with different parameter settings. Inter-ensemble represents the combination of different models or different intra-ensembles.

In this section, we describe our methods to address the task of entity recognition in EHRs. We consider entity recognition as a sequence labeling task whose goal is to assign one of B (Beginning), I (Inside), or O (Outside) tags to each word. As illustrated in Figure 1, we firstly preprocessed EHRs using sentence segmentation and tokenization. We then implemented feature-based and neural network-based models to detect ADEs and related medications.

### Data preprocessing

We applied two natural language processors to the EHRs: the LingPipe sentence splitter^31^ and the OSCAR4 tokenizer.^32^ Because EHRs usually contain noisy text, such as deidentified terms, special symbols, texts in tables and random new lines, we revised the original text as well as the output of the sentence splitter and the tokenizer to obtain better sentences and tokens. Specifically, we replaced all deidentified terms with the static string “DEIDTERM” (eg, both “[**Known lastname 3234**]” and [**2115-2-22**]” are converted to DEIDTERM). We then implemented two rules to postprocess the resulting sentences and tokens. Since the sentence splitter ignores new line characters (“\n”) when segmenting sentences, we further split sentences containing any of the following strings: “\n\n,” “:\n,” or “]\n.” Tokens containing any of the following special characters were broken into fine-grained tokens: @, *,? , ∼, %,) and (.

### Feature-based CRF model

For the feature-based model, we used NERSuite^33^ with three different groups of features. The first group consists of token-based features corresponding to orthographic, lexical, and syntactic information. For orthographic features, we used word shape in which all uppercase letters were converted to “A,” all lowercase letters were converted to “a,” all digits were converted to “#,” and other symbols were retained. Lexical–syntactic features were obtained using the GENIA tagger.^34^ The second group consists of dictionary features generated using a disease list obtained from the Human Disease Ontology^35^ and a list of ADE terms. The list of ADE terms was compiled from MedDRA side effects,^9^ the Ontology of Drug Adverse Events,^36^ and adverse reaction entities were extracted from the TAC 2017 Shared Task corpus.^13^ The coverage of these dictionaries is listed in Supplementary Table 5 of the Appendix. The third group includes cluster features inspired by Lance et al.^37^ To produce word clusters, we used word2vec^38^ to train word vectors on 59 652 discharge summaries of MIMIC III.^5^ We varied the window sizes (2 and 5) and the number of clusters (128, 256, and 512) following Lance et al.^37^ We also tried 1024 clusters, but the performance was not improved.

### NN-based model

**Figure 2.**
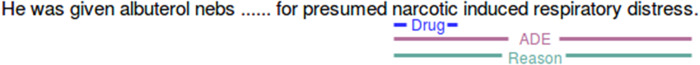
An example of nested entities.

We implemented the NN model proposed in Ju et al,^28^ which extracts both nested and flat (non-nested) entities without using any linguistic features or external knowledge. As an example of nested entities, consider Figure 2, where the *Drug* entity is embedded (nested) inside *Reason* and *ADE*. In addition, *ADE* and *Reason* are polysemous entities, since they both cover the same text span; we treat these as a special case of nested entities.

**Figure 3.**
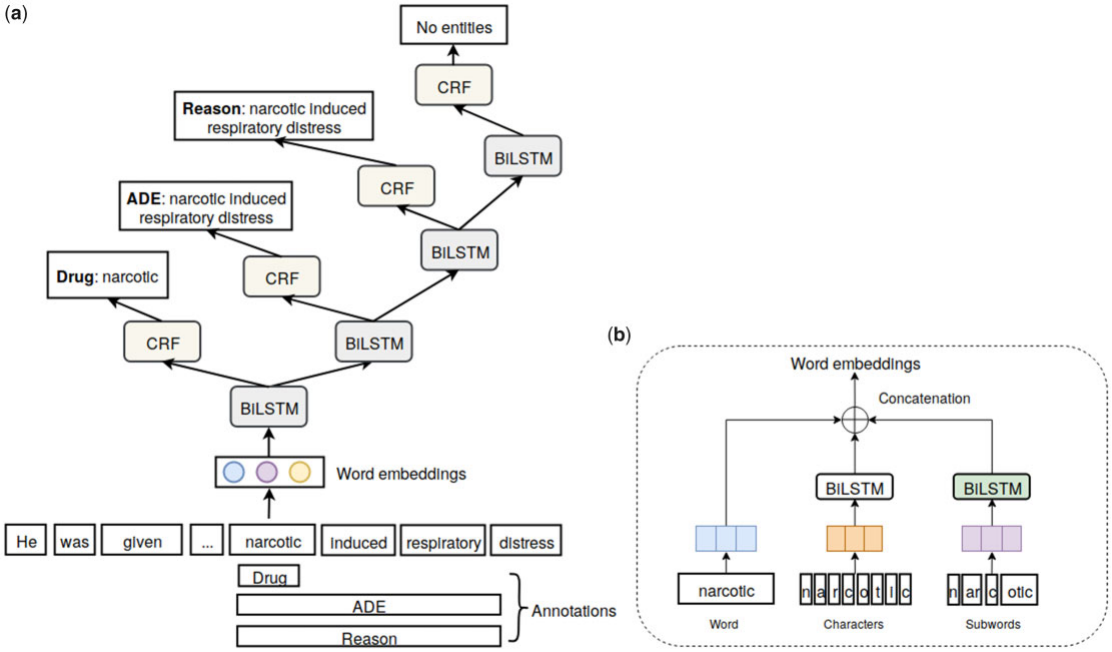
The architecture of the neural model. (a) is the model architecture while (b) is the composition of the word embeddings.

The architecture of the model, which dynamically extracts nested entities in an inside-outside (innermost-outermost) order, is depicted in Figure 3. Specifically, it firstly maps each word to a vector (ie, word embedding) by looking up the corresponding pretrained word embeddings. To capture orthographic features, character embeddings are concatenated with word embeddings and serve as the input of a BiLSTM to generate context features. On top of the BiLSTM layer, a CRF layer is introduced to predict word labels based on the maximum joint probability of the input word sequence. In essence, the BiLSTM and CRF layers together constitute a flat named entity recognition (NER) layer. When entities are nested, the inner entities can provide informative clues for the detection of outer entities. To make use of such information, the model automatically stacks flat NER layers for outer entities on top of the inner entity recognition layer. The model directly takes the corresponding context representation produced by the flat NER layer for each nonentity word. For each detected entity, the model automatically averages the context representation of words in the entity to encode inner entity information as well as facilitating outer entity detection. Then, it automatically stacks flat NER layers until no further entities are detected, which enables both inner and outer entity detection in a dynamic manner. As a result, the number of flat NER layers depends on the degree of nestedness of entities contained in the input word sequences. The dynamic nature of our model enables us to extract polysemous entities by stacking flat NER layers to recognize other categories with the same text span.

Table 1 provides statistics regarding the training data. We observe that there are many rare and unknown words (words that are unseen in the training data) included in entities, which makes their extraction challenging. To address this problem, we used byte pair encoding^29^ that represents words by iteratively merging the most frequent adjacent/consecutive characters into longer character sequences (ie, subwords). We collected all the words occurring in the training data and iteratively combined the most frequent pairs of neighboring characters or character sequences, resulting in a tokenization model in which each line contains one subword coupled with its unique id. The tokenization model was used to split word sequences into subword sequences that may carry patterns of informative words in entities. We then concatenated the subword embeddings with word embeddings which were used as input to our model.

**Table 1.** Statistics of the data set. Rare words are words that occur only once in the data. Unknown words refer to words that are not seen in the training set

Item	Training	Development
Document	242	61
Entities	41 171	9776
Nest level 1 entity (flat entities)	41 109	9760
Nest level 2 entity	61	16
Nest level 3 entity	1	0
Polysemous entity	47	13
Textually nested entity	15	3
ADE	785	174
Dosage	3401	820
Drug	13 109	3114
Duration	499	93
Form	5340	1311
Frequency	5075	1205
Reason	3105	750
Route	4479	996
Strength	5378	1313
Unknown words /Unique words	–	17.00%
Rare words /Unique words	37.19%	37.69%
EUNKs/All entities	–	2.67%
ERAREs /All entities	1.89%	3.88%

Abbreviations: ADE, adverse drug event; EUNKs, entities that contain unknown words, ERAREs, entities that contain rare words.

### Experimental design

The 2018 n2c2 Shared Task Track 2 provided 505 annotated discharge summaries extracted from MIMIC III, of which 303 were released for training and 202 were used for testing. The statistics are shown in Table 1. To determine the best ensemble setting, we further divided the training set into two subsets: 80% for training and 20% for development; the latter is used to validate the models. We evaluated all models using lenient metrics in terms of precision, recall, and F-score, which were the main ones used in Track 2. According to lenient metrics, a predicted entity is considered to be correct if its category is correct, and its span partially overlaps with that of the gold standard entity.

Regarding the CRF model, since lexical and syntactic features are default input features of NERSuite, we treated them as baseline features and evaluated the combinations of the remaining features (ie, word shape, dictionary, and cluster features). For the NN models, we experimented with following settings:

1. *baseline* model: using word embeddings concatenated with character embeddings as the input to the neural layered model. We excluded part-of-speech and dictionary features from the baseline model because experimental results showed that those features slightly harmed the performance. We randomly initialized a vector for each character. Given a word, we fed its character sequence to a BiLSTM and concatenated the bidirectional last hidden states as the character embeddings.
2. *csub* model: using subword embeddings and character embeddings as the input to the model. Similarly to character embeddings, we used a different BiLSTM to obtain subword embeddings. We used varying vocabulary sizes of [300; 1000; 4000; 8000; 16 000] to train different tokenization models. As a result, we generated five different versions of subword sequences for a given input word sequence. An example is shown in Supplementary Table 6 of the Appendix. Each different version of subword sequences produced subword embeddings, which were individually used in the model. Instead of predicting label sequences at the word level, we predicted the label for each word at the subword level. When merging the subword labels into their corresponding word labels, we kept the first subword label as their word label. Taking the entity “vincristine toxic polyneuropathy” as an example, we selected one version of the tokenization model to generate its subword sequence “-v, in, c, r, ist, ine, -toxic, -poly, ne, u, rop, athy” where “-” represents a whitespace character. Using the *csub* model, the predicted subword-level label sequence is [B-ADE, I-ADE, I-ADE, I-ADE, I-ADE, I-ADE, I-ADE, I-ADE, I-ADE, I-ADE, I-ADE, I-ADE, I-ADE], while the corresponding word-level label sequence is [B-ADE, I-ADE, I-ADE]. When merging subword-level labels for each word, we picked up the first subword label (eg, “B-ADE”) among subword labels and attached it to the word “vincristine” as the final word-level label.
3. *wsub* model: using the concatenation of word embeddings and each version of the subword sequences obtained from 2 as the input to the model.
4. *wcsub* model: using the concatenation of baseline embeddings (ie, word and character embeddings) and each version of the subword sequences obtained from 2 as the input to the model.

By combining these settings, we have 16 models in total.

We produced different combinations of intra- and inter-models using the majority voting method.^30^ Specifically, we merged predictions from: (1) different feature combinations of the CRF models, (2) combinations of NN baseline and the remaining NN models, which were internally ensembled with vocabulary sizes; and (3) all of the previously mentioned settings. We selected entities that have the most votes for their specific span.

## RESULTS

The following experimental results were calculated using our development set and the official test set. Table 2 summarizes lenient performance of the CRF and NN models, including their combinations, on the development set. The NN-based models were tuned using Bayesian optimization.^40^ The best hyper parameter values are listed in Table 7 in the Appendix. Please refer to Supplementary Table 8 in the Appendix for detailed performances. As shown in Table 2, using word shape (ws) or dictionary features (df) alone reduced the CRF performance, whereas their combination produced the highest lenient precision. Our CRF model achieved the best lenient F-score when using only cluster features (cf) and the highest recall when further combined with df.

**Table 2.** Performance of CRF and NN models on the development set. For each model, the best lenient metrics of precision, recall, and F-score are shown in bold

Model	Precision	Recall	F-score
**CRF**			
Baseline (Lexical and syntactic features)	0.9525	0.8825	0.9162
Baseline + word shape (ws)	0.9527	0.8815	0.9157
Baseline + dictionary features (df)	0.9511	0.8829	0.9157
Baseline + cluster features (cf)*	0.9504	0.8902	**0.9193**
Baseline + ws + df	**0.9523**	0.8821	0.9158
Baseline + ws + cf	0.9491	0.8898	0.9185
Baseline + df + cf	0.9494	**0.8903**	0.9189
Baseline + ws + df + cf	0.9486	0.8900	0.9184
**Neural Network**			
Baseline (word + characters)	0.9476	0.8995	0.9230
Csub (characters + subword)	**0.9502**	0.9042	0.9266
Wsub (word + subword)	0.9496	0.9044	0.9264
Wcsub (word + subword + characters)*	0.9498	**0.9066**	**0.9277**
**Ensemble**			
Inter-CRF	0.9466	0.8935	0.9193
Intra-csub	**0.9656**	0.8981	0.9306
Intra-wsub	0.9638	0.9013	0.9315
Intra-wcsub	0.9641	0.9010	0.9315
Inter-NN	0.9591	0.9084	**0.9331**
NN-CRF	0.9401	**0.9209**	0.9304

* represents significance value at *P* < .05 with approximate randomization significance test.^39^

Abbreviations: CRF, conditional random fields; NN, neural network.

Compared with the CRF models, our NN models obtained consistent improvements in terms of lenient recall and F-score. We obtained the best performance with the *wcsub* model, which employs the embeddings of words, subwords, and characters. The removal of word embeddings yielded the best precision without significantly sacrificing recall, thus achieving comparable lenient F-score. The introduction of subwords to each individual character or word embedding produced better performance than their combination (ie, the NN *baseline* model).

Ensemble of models outperformed their individual ones except the inter-CRF. We obtained the best lenient F-score when externally combining each intra-NN model, whereas the best recall was produced using an ensemble of inter-CRF and inter-NN models (ie, NN-CRF).

Table 3 shows the performance of two ensemble settings on the test set. The first setting was our submission setting, which was an ensemble of inter-CRF, intra-csub, intra-wsub, and *wcsub* models (initialized only with vocabulary sizes of 1000 and 4000). Using this setting, our ensemble model performed well in predicting entity types such as *Strength* and *Frequency*. The second setting was the inter-NN model setting, which produced the best lenient F-score. However, it was not selected for submission to the shared task due to time limitations. We refer readers to Supplementary Table 9 in the Appendix for details.

**Table 3.** Lenient performance on the test set with submission and inter-NN settings

Entity type	Precision	Recall	F-score
**Submission Setting**			
Strength	0.9815	0.9804	0.9810
Frequency	0.9788	0.9666	0.9727
Route	0.9662	0.9445	0.9552
Drug	0.9567	0.9533	0.9550
Form	0.9653	0.9436	0.9543
Dosage	0.9356	0.9433	0.9395
Duration	0.8875	0.7513	0.8138
Reason	0.7254	0.5470	0.6237
ADE	0.4697	0.1984	0.2790
Overall (micro)	0.9444	0.9073	0.9255
**Inter-NN Setting**			
Overall (micro)	0.9599	0.8979	0.9278

Abbreviation: ADE, adverse drug event.

## DISCUSSION

We conducted an error analysis of predictions on the development set for the best individual and ensemble models. We divided false positive entities into two classes: (1) category error (CE), corresponding to entities that have correct lenient spans but incorrect categories; and (2) span error (SE), corresponding to entities that have both incorrect spans and categories.

**Figure 4.**
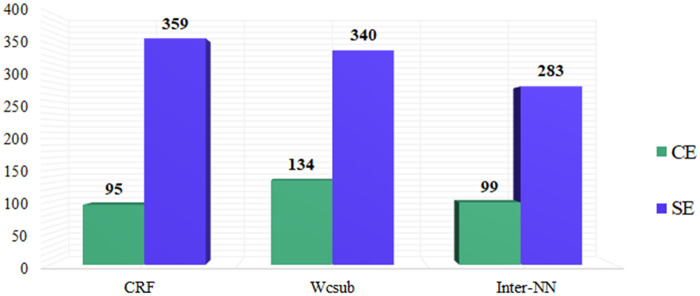
Statistics of CEs and SEs for our best individual and ensemble models on the development set.

Figure 4 shows the statistics of CEs and SEs for our best individual and ensemble model on the development set. In general, our *wcsub* model produced more CEs than the CRF model, indicating that the *wcsub* model detected more informative text regions. One reason is that our *wcsub* model uses word embeddings, which encode denser context features than the hand-crafted feature set used in the CRF model, thus leading to enhanced detection of relevant text regions. Another reason is that our CRF model only handles flat entities (ie, it can only detect outermost entities), and it can only predict a single category for each of these entities. However, there are many entities that have multiple categories (eg, *ADE* and *Reason* entities), which are additionally utilized during training of our *wcsub* model. Furthermore, we incorporated subwords to enhance representation of sparse entities that were missed by the baseline model. When combining the predictions from all NN models, the number of errors (ie, CEs and SEs) reduced, demonstrating the important contribution of ensemble predictions.

**Figure 5.**
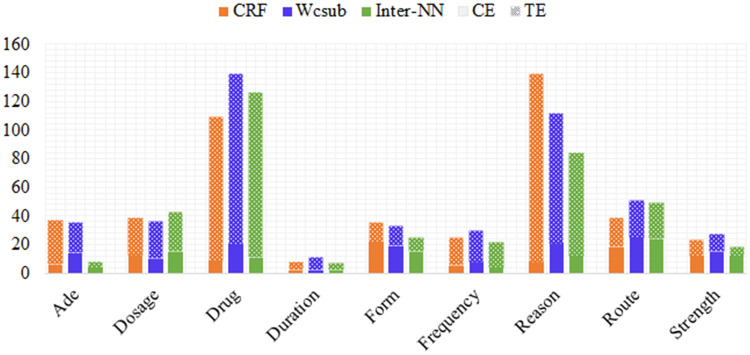
Statistics of CEs and SEs for each category on the development set.

We further analyzed the distribution of CEs and SEs for each category, as shown in Figure 5. All of the models yield the fewest SEs for *Duration* entities and the most for *Drug* and *Reason* entities. This is probably because *Duration* entities have common patterns (eg, most of them include informative words such as “day(s),” “for,” etc within their sentential context). In contrast, *Drug* and *Reason* entities are more variable and require contextual information from neighboring sentences. For example, consider the following extract: “It was felt that an injection would not be helpful, given the diffuse nature of his headaches. Fioricet was discontinued, as it did not appear to be effective anymore.” Here, “headaches” is a *Reason* entity and “Fioricet” is a *Drug* entity. Information from the second sentence is needed to tag “headaches” in the first sentence as *Reason*, whereas the reverse is true to facilitate the accurate categorization of “Fioricet” in the second sentence. However, such information is unavailable in our models as they only consider sentence level information. Based on the annotation guidelines, a *Reason* entity is only annotated when it is attached to a *Drug* name. Therefore, the misclassification and mis-extraction of *Drug* names result in an increase in CEs and SEs for *Reason* entities. Moreover, many *Reason* entities contain unknown or rare words, which inevitably limits the ability of our models to recognize them. Apart from *Reason* and *Drug*, *Route* also has a high number of CEs since most *Route* entities are confused with either *Drug* or *Form* entities (eg, “Inhalation” can be either *Form* or *Route*). Such confusion further contributes to the high CEs in *Drug* and *Form* categories.

**Figure 6.**
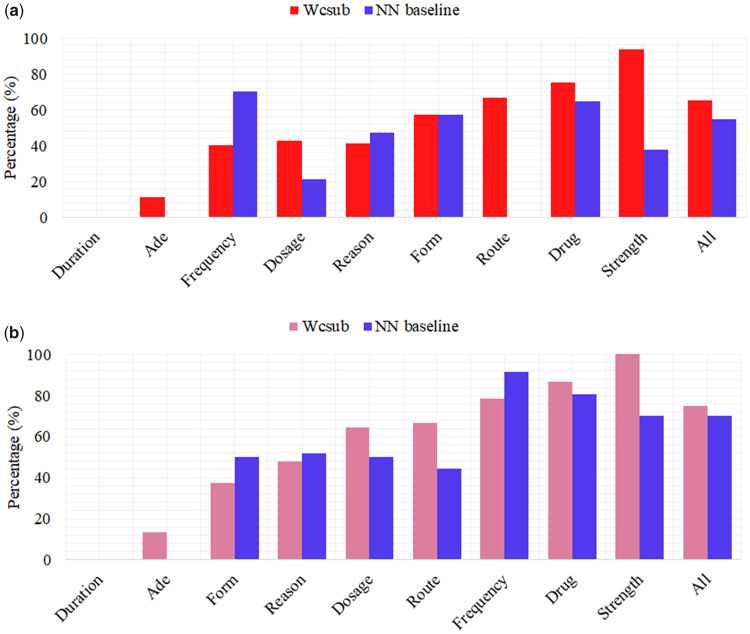
Percentage of category-wise extracted EUNKs (a) and ERAREs (b).

Figure 6 shows the percentages of EUNKs (entities that contain unknown words) (a) and ERAREs (entities that contain rare words) (b) extracted for each category, respectively. As shown in Figure 6a, our *wcsub* model is better able to extract EUNKs than the NN baseline model. This result demonstrates that, for most categories, the incorporation of subword information helps the *wcsub* model to recognize such entities more accurately. Among all categories, our *wcsub* model achieved the highest improvement for *Strength* entities. Entities in this category commonly include words that exhibit a specific pattern within them (ie, “digits symbol digits” eg, “150(2”); the incorporation of subword features in the *wcsub* model means that it can help to take such internal features of words into account.

For *Frequency* entities, the *wcsub* model misclassified three instances as *Duration*, since they are coupled with time units: “pm,” “am,” and “hs.” Both of our models exhibit comparable performance for the *Duration* and *Form* categories, whose entities are often composed of informative words, such as “day,” “month,” “tablet,” etc. In terms of *Reason*, the *wcsub* model extracted two fewer entities than the baseline, which correspond to long phrases such as “patchy infiltrates concerning for biliary sepsis.” However, it was able to additionally extract a number of shorter entities (eg, “maculopapular rash”), which were missed in predictions from the NN baseline model. In contrast to *Reason*, the *wcsub* model increases the recall of *Drug* entities, which constitute the largest proportion of all entities.

We additionally analyzed how using subword units can improve the extraction of entities containing rare words; the results are shown in Figure 6b. It can be seen that the *wcsub* model improves the recall of sparse entities, especially for those belonging to *Strength*, *Dosage*, and *Route*. This phenomenon indicates that entities with certain patterns (eg, real values followed by units) benefit significantly from the use of subwords. In contrast, however, our *wcsub* model fails to capture sparse entities belonging to the categories of *Reason*, *Form*, and *Frequency.* This is likely to be because they require contextual information beyond the sentence level.

Table 4 lists the fine-grained matching statistics of our models for lenient evaluation. On the development set, all models are able to accurately label exact text spans with correct categories (see *Strict* results). The second most frequent type of match is “Includes” (ie, predictions have wider spans than their corresponding gold standard annotations). Among those predictions, the *Frequency* and *ADE* categories have the highest and lowest occurrences, respectively. In contrast to *Frequency*, the scarce occurrences of *ADEs* accounts for their lowest number among all categories. In the case of “Is included” (ie, predictions have narrower spans than their corresponding gold standard annotations), *Drug* entities account for the largest portion, whereas *Route* and *ADE* entities have the lowest numbers. Problems with *Drug* entities mainly concern the inconsistent inclusion of symbols in the annotated span (eg, “]” is excluded in the span “fluticasone [flonase]”) which confused our models in terms of determining the correct span. For the *Route* category, there is only one incorrect span prediction, in which “IV” was extracted as the entity span, although it should also have included its neighboring words. Similarly, there is only one *ADE* annotation that exhibits the same phenomenon (ie, “somnolent” was predicted instead of “completely somnolent.” All models predict only a very small number of spans that partially overlap with the gold standard annotations.

**Table 4.** Fine-grained lenient matching statistics of individual and ensemble models on the development set

Matching type	CRF (%)	Wcsub (%)	Inter-NN (%)	NN-CRF (%)
Strict	95.73	95.02	95.62	95.51
Includes	2.71	3.17	2.83	2.75
Is included	1.53	1.76	1.50	1.69
Partial overlap	0.03	0.05	0.06	0.06

## CONCLUSION

We have described an ensemble of neural models that enable automatic extraction of medications and their attributes from EHRs. The neural model is capable of recognizing nested entities where the nesting may consist of either additional embedded entities or polysemous entities in the same text span with an alternative semantic type, by passing the representation encoding inner entities to subsequent layers to improve outer entity recognition. Furthermore, we utilized subwords to improve the representation of rare and unknown words contained within entities. We additionally implemented a CRF model that uses various features including token-based features, dictionary features, and cluster features to extract flat entities. Compared to the CRF model, our neural model automatically learns high and abstract level features from the data, thus removing the dependence on any external knowledge resources or hand-crafted linguistic features. Combining predictions from different settings of the neural model boosts the performance. However, combining predictions from NN and CRF models demonstrates few advantages since the NN models have superior performance.

In addition to extracting drug-related information, our models could be readily extended to extract other types of medical entities, such as disease and risk factors, without requiring extra human effort. Our results indicate that our NN models are able to detect sparse nested entities to a high degree of accuracy. This paves the way for the automated extraction of fine-grained entity information to enhance understanding of EHRs (eg, drug history for individual patients and disease-specific adverse drug effects). Furthermore, the detection of most entity categories, especially those containing sparse entities, benefits from the use of subword information. The recognition of these sparse entities is of great importance to the development of the clinical domain to aid tasks such as drug discovery and disease recognition.

In future work, we will extend our method to improve the recognition of ADEs by integrating advanced neural network models and additional corpus resources. We will also apply our method to extract different types of medical entities including both flat and nested entities. This will reinforce the importance of our method in helping to detect fine-grained information, thus providing a feasible and effective approach toward medical information extraction.

## FUNDING

This research was supported by EMPATHY (Grant ID: BB/M006891/1) and MMPathIC (Grant ID: MR/N00583X/1).

## AUTHOR CONTRIBUTIONS

MJ implemented neural network-based models, ran experiments, and conducted analysis of their outputs. NN processed the data and implemented the CRF-based model and the majority voting method. MM and SA supervised all steps of the work. MJ and NN drafted the manuscript. MM and SA revised the manuscript. All authors read and approved the final version of the manuscript.

## Supplementary Material

ocz075_Supplementary_Data
