# Supplementary material for: An ensemble of neural models for nested adverse drug events and medication extraction with subwords
Source: J Am Med Inform Assoc. 2019 Jun 14;27(1):22–30. doi: 10.1093/jamia/ocz075 (PMC6913208; doi:10.1093/jamia/ocz075)
Supplement: ocz075_Supplementary_Data [file ocz075_supplementary_data.docx]

# **APPENDIX**

Table 5 presents the coverage percentage between our dictionaries and data sets. Table 6 shows an example of five versions of subword sequences generated by different tokenisation models. We report the best hyper parameter values on the development set in Table 7. Table 8 describes the performances of our each individual NN-based model and ensemble models. Table 9 lists the performances of strict evaluation on the test set.

**Table 5.** Entity coverage percentage between data set and ontologies.

| **Ontology** | **Entity** | **Train**  **(%)** | **Development**  **(%)** | **Test**  **(%)** | **Development - Train (%)** | **Test – Train (%)** |
| --- | --- | --- | --- | --- | --- | --- |
| Human Disease Ontology | All | 2.04 | 1.98 | 2.07 | 3.94 | 2.55 |
|  | Unique | 3.29 | 3.65 | 3.24 | 2.74 | 2.27 |
| MedDRA side effects | All | 3.33 | 3.71 | 3.57 | 5.41 | 4.8 |
|  | Unique | 4.35 | 5.22 | 4.95 | 4.32 | 3.89 |
| Ontology of Drug Adverse Event | All | 2.81 | 2.93 | 2.86 | 1.11 | 1.31 |
|  | Unique | 1.83 | 2.78 | 1.91 | 1.01 | 0.77 |
| TAC entities | All | 4.15 | 4.10 | 4.23 | 3.32 | 3.09 |
|  | Unique | 4.90 | 5.67 | 4.76 | 3.17 | 2.35 |
| Overall | All | 4.91 | 5.10 | 5.12 | 8.61 | 6.48 |
|  | Unique | 7.56 | 8.61 | 7.8 | 6.77 | 5.69 |

**Table 6.** Five versions of subword sequences for the given *ADE* entity “vincristine toxic polyneuropathy” that contains a *Drug* entity “Vincristine” inside itself. “▁” represents the whitespace.

| **Vocabulary size of the tokenisation model** |  | **Subword sequence** |
| --- | --- | --- |
| 300 |  | ▁v, in, c, r, ist, ine, ▁to, x, ic, ▁p, o, ly, ne, u, ro, p, at, h, y |
| 1000 |  | ▁v, in, c, r, ist, ine, ▁to, x, ic, ▁po, ly, ne, u, rop, at, hy |
| 4000 |  | ▁v, in, c, r, ist, ine, ▁toxic, ▁poly, ne, u, rop, athy |
| 8000 |  | ▁v, inc, rist, ine, ▁toxic, ▁poly, ne, uropathy |
| 16000 |  | ▁vincristine, ▁toxic, ▁polyneuropathy |

**Table 7.** Best hyper parameters of individual NN models.

| **Value range** | Batch size | Learning rate | Weight decay | Dropout | Gradient clipping | Vocab size |
| --- | --- | --- | --- | --- | --- | --- |
|  | [16-256] | [0.001-0.02] | [1e-08, 0.001] | [0.1-0.9] | [5, 50] | [300,1,000, 4,000, 8,000, 16,000] |
| Baseline | 144 | 0.007240 | 2.73e-4 | 0.419363 | 11 | - |
| Csub | 255 | 0.006715 | 1.08e-4 | 0.429264 | 28 | 4,000 |
| Wsub | 224 | 0.008162 | 8.28e-05 | 0.475458 | 11 | 4,000 |
| Wcsub | 240 | 0.004740 | 1.32e-05 | 0.513470 | 47 | 300 |

**Table 8.** Performances of individual NN models and intra- and inter- ensembling models on the development set.

| **Model** | **Vocab Size** | **Ensemble** | | | | | |
| --- | --- | --- | --- | --- | --- | --- | --- |
|  |  | **Strict** | | | **Lenient** | | |
|  |  | **Precision** | **Recall** | **F-score** | **Precision** | **Recall** | **F-score** |
| Baseline | - | 0.8935 | 0.8675 | 0.8803 | 0.9484 | 0.9038 | 0.9256 |
| Csub | 300 | 0.8892 | 0.8649 | 0.8768 | 0.9472 | 0.9032 | 0.9247 |
|  | 1000 | 0.8895 | 0.8769 | 0.8832 | 0.9439 | 0.9144 | 0.9289 |
|  | 4000 | 0.8890 | 0.8831 | 0.8860 | 0.9454 | 0.9185 | **0.9317** |
|  | 8000 | 0.8953 | 0.8762 | 0.8856 | 0.9493 | 0.9109 | 0.9297 |
|  | 16000 | 0.8824 | 0.8673 | 0.8748 | 0.9406 | 0.9061 | 0.9230 |
|  | Ensemble | 0.9218 | 0.8610 | 0.8904 | 0.9656 | 0.8981 | **0.9306** |
| Wsub | 300 | 0.8906 | 0.8710 | 0.8807 | 0.9486 | 0.9095 | 0.9286 |
|  | 1000 | 0.8912 | 0.8750 | 0.8830 | 0.9463 | 0.9124 | 0.9291 |
|  | 4000 | 0.8895 | 0.8776 | 0.8835 | 0.9422 | 0.9146 | 0.9282 |
|  | 8000 | 0.8891 | 0.8815 | 0.8853 | 0.9458 | 0.9156 | 0.9305 |
|  | 16000 | 0.8758 | 0.8782 | 0.8770 | 0.9385 | 0.9146 | 0.9264 |
|  | Ensemble | 0.9198 | 0.8634 | 0.8907 | 0.9638 | 0.9013 | **0.9315** |
| Wcsub | 300 | 0.8909 | 0.8783 | 0.8846 | 0.9449 | 0.9153 | 0.9299 |
|  | 1000 | 0.8864 | 0.8784 | 0.8824 | 0.9461 | 0.9125 | 0.9290 |
|  | 4000 | 0.8897 | 0.8810 | 0.8853 | 0.9453 | 0.9183 | **0.9316** |
|  | 8000 | 0.8889 | 0.8744 | 0.8816 | 0.9467 | 0.9115 | 0.9288 |
|  | 16000 | 0.8892 | 0.8754 | 0.8823 | 0.9439 | 0.9121 | 0.9277 |
|  | Ensemble | 0.9210 | 0.8637 | 0.8915 | 0.9641 | 0.9010 | **0.9315** |
| Inter-NN | Ensemble | 0.9105 | 0.872 | 0.8909 | 0.9591 | 0.9084 | **0.9331** |
| NN-CRF | Ensemble | 0.8884 | 0.8838 | 0.8861 | 0.9423 | 0.9162 | 0.9291 |

**Table 9.** The performances of our submission in terms of strict precision, recall and F-score on the test set.

| **Entity Type** | **Precision** | **Recall** | **F-score** |
| --- | --- | --- | --- |
| Drug | 0.9137 | 0.9342 | 0.9238 |
| Strength | 0.9424 | 0.9629 | 0.9525 |
| Duration | 0.7568 | 0.6667 | 0.7089 |
| Route | 0.9479 | 0.9331 | 0.9405 |
| Form | 0.9296 | 0.9147 | 0.9221 |
| Ade | 0.4491 | 0.1904 | 0.2674 |
| Dosage | 0.8984 | 0.9168 | 0.9075 |
| Reason | 0.6494 | 0.5014 | 0.5659 |
| Frequency | 0.8233 | 0.8445 | 0.8338 |
| Overall (micro) | 0.8890 | 0.8722 | 0.8805 |
| Overall (macro) | 0.8854 | 0.8599 | 0.8712 |
